# Editorial: The gut microbiota and brain interactions in healthy and pathological circumstances: From understanding to therapy

**DOI:** 10.3389/fnins.2022.1052350

**Published:** 2022-10-17

**Authors:** El Hiba Omar, Tiziano Balzano, El Hidan Moulay Abdelmonaim, Arumugam R. Jayakumar

**Affiliations:** ^1^Faculty of Sciences, University Chouaib Doukkali, EL Jadida, Morocco; ^2^HM CINAC (Centro Integral de Neurociencias Abarca Campal), Hospital Universitario HM Puerta del Sur, HM Hospitales, Madrid, Spain; ^3^Faculty of Applied Sciences, University Ibn Zohr, Agadir, Morocco; ^4^Department of Obstetrics, Gynecology and Reproductive Science, Leonard M. Miller School of Medicine, University of Miami School of Medicine, Miami, FL, United States

**Keywords:** gut microbiota, central nervous system, brain disease, multiple sclerosis, alcohol use disorders, Parkinson's disease, dysbiosis, probiotic

The gut microbiota (GM) is a complex collection of microorganisms that includes bacteria and other microbes such as fungi, archaea, viruses, and protozoans, that produce energy from digested food, regulate immune function and protect against pathogens (Alander et al., 1999). Microbiological studies have estimated 100 trillion bacteria in an adult's body, while 80% of which exist in the gut (de Vos and de Vos, 2012; Lozupone et al., 2012). Studies have shown that changes in GM could affect the brain's physiological, behavioral, and cognitive functions (Heijtz et al., 2011; Mayer et al., 2014; Jenkins et al., 2016). Accordingly, examination of the interaction between GM and the brain (gut-brain axis, bidirectional) have gradually increased in recent years (Honarpisheh et al., 2022; Liu et al., 2022; Palepu and Dandekar, 2022; Ribeiro et al., 2022; Wang et al., 2022).

Experimental studies suggest that the microbiome strongly influences the brain by altering the blood-brain barrier, neurochemical, neuroimmune/inflammatory, and neuroendocrine systems, as well as by stimulating central nervous system (CNS) amino acids and neurotransmitters imbalance. However, underlying the molecular events and the impact of GM on various neuropathogenesis remains poorly understood. The present issue, therefore, targets the impact of GM on CNS complications.

The current Research Topic contains six articles with two reviews and four original research articles emphasizing the interaction between GM and the brain in various pathological conditions, and more recent progress in GM effects on neurobehavioral/ cognitive processes. These Research Topics also discuss recent development in identifying therapies to treat defective brains and associated diseases due to GM influences on the brain.

In that context, Duarte-Silva et al. reviewed recent findings on the role of microbial-derived molecules in the pathogenesis and treatment of multiple sclerosis by focusing on short-chain fatty acids (SCFAs), polyamines, and urolithins. Li et al. described the pathogenesis and the possible treatment strategies of AUD-induced cognitive deficits, anxiety, depression, and intestinal flora imbalance. The authors provide novel mechanistic insights and therapeutic options to treat alcohol use disorders (AUD)-induced neuropsychiatric disorders where GM plays an important role. These articles collectively offer crucial information on GM's effect on CNS disorders.

Four other articles are original research that has provided insightful and pertinent clinical and epidemiological data on GM-related neurodegenerative disorders and aging-related pathologies, as well as an experimental study on neurobehavioral/cognitive deficits due to GM dysbiosis.

The Research Topic by Kenna et al. explored the role of GM in Parkinson's disease (PD) by combining 16S rRNA sequencing and functional predictions of alterations in host metabolic pathways in a larger multicenter Australian cohort. This topic provides extremely useful information on the gut-brain axis and potential targets for future therapy to alleviate GM-mediated CNS complications.

Wang et al. provide a piece of amazing information that present a better understanding of the prevalence of constipation in patients with dementia and mild cognitive impairment subtypes and explores the association between constipation and cognitive dysfunction. Interestingly, Rahimlou et al. document that supplementation with probiotics has beneficial effects on serum levels of various factors associated with systemic inflammation in patients with multiple sclerosis. Additionally, Tamada et al. provided experimental evidence of cognitive flexibility in mice with GM dysbiosis *via* a novel cognitive flexibility test protocol using a touch screen operating system.

In conclusion, the article collection of the current Research Topic provides novel and up-to-date insightful clinical, epidemiological, and experimental data, as well as a literature overview on the involvement of GM in neurobehavioral/cognitive deficits and the pathophysiology of different brain disorders. The Research Topic covered on neurodegenerative diseases and on the aging post-GM impact on CNS, together with the explored therapeutic potential of probiotics and various agents, we believe will attract not only scientists but also general readers who warrant more information on gut-brain axis-related CNS diseases.

## Author contributions

All authors listed have made a substantial, direct, and intellectual contribution to the work and approved it for publication.

## Funding

This work effort was supported by JUAN DE LA CIERVA-INCORPORACIÓN (IJC2020-043918-I), MCIN/AEI/10.13039/501100011033 and by European Union NextGenerationEU/PRTR (TB).

## Conflict of interest

The authors declare that the research was conducted in the absence of any commercial or financial relationships that could be construed as a potential conflict of interest.

## Publisher's note

All claims expressed in this article are solely those of the authors and do not necessarily represent those of their affiliated organizations, or those of the publisher, the editors and the reviewers. Any product that may be evaluated in this article, or claim that may be made by its manufacturer, is not guaranteed or endorsed by the publisher.
